# Biochemical and Biophysical Properties of Extracellular Matrix Nanofibers Modulate iPSC-Derived Human Hepatocyte Maturation

**DOI:** 10.1002/jbm.a.37998

**Published:** 2025-10

**Authors:** Yang Yuan, Liszt C. Madruga, Kristen Y. Cotton, Matt J. Kipper, Salman R. Khetani

**Affiliations:** 1 Department of Biomedical Engineering, University of Illinois Chicago, Chicago, Illinois, USA; 2 Department of Chemical and Biological Engineering, Colorado State University, Fort Collins, Colorado, USA

**Keywords:** definitive endoderm, electrospinning, hydrogel, topography, YAP

## Abstract

Human liver models grown in the lab are used for testing drug metabolism and toxicity, studying liver diseases, and developing new therapies. Induced pluripotent stem cell (iPSC)-derived hepatocyte-like cells (HLCs) provide a renewable alternative to scarce primary human hepatocytes (PHHs), but they remain functionally immature compared to adult liver cells. The extracellular matrix (ECM) is a key regulator of liver cell behavior, yet how its biochemical makeup, stiffness, and structural organization work together to influence HLC maturation is not well understood. Here, we engineered electrospun nanofibers from collagen I, chitosan, porcine liver ECM (PLECM), and blends of these materials. Over 3 weeks of differentiation, HLCs cultured on ECM nanofibers showed more advanced functional maturation than those grown on standard Geltrex-coated substrates. Importantly, chitosan/collagen nanofibers promoted greater HLC function than either hydrogels of similar stiffness or proteins adsorbed to glass, highlighting the importance of nanoscale topography. By contrast, stiffer polyvinyl alcohol nanofibers of comparable size failed to enhance HLC maturation, a result linked to higher nuclear activity of the mechanosensor Yes-associated protein 1 (YAP). These findings demonstrate that ECM nanofibers drive more mature iPSC-HLCs and advance the development of predictive human liver models for drug discovery, disease modeling, and regenerative medicine.

## 1 ∣ Introduction

Bioengineered liver models have been developed to replicate the structural and functional complexity of native liver tissue, offering significant potential for drug testing and disease modeling. The two fundamental components of bioengineered liver models are hepatic cells and the supportive scaffold, which are pivotal in maintaining a stable liver phenotype. The selection of appropriate cell types is crucial; while primary human hepatocytes (PHHs) are ideal due to their mature characteristics, their scarcity and dedifferentiation limit routine drug screening [1, 2]. In contrast, induced pluripotent stem cell-derived hepatocyte-like cells (iPSC-HLCs) serve as an almost unlimited cell source. However, when differentiated using conventional protocols on tissue culture plastic or glass plates coated with extracellular matrix (ECM) proteins, these cells display a fetal-like phenotype [3]. Therefore, generating a physiologically relevant scaffold biomimetic to liver ECM is crucial for enhanced cell differentiation and maturation.

ECM constitutes a small fraction of the liver's composition, accounting for less than 3% of the liver area within a tissue section. Still, it is crucial in maintaining hepatocyte function, polarity, and differentiation [4]. Previously, the differentiation of HLCs on 2D culture formats involved materials such as fibronectin [5], Matrigel [6-8], and rat tail collagen type I [9], combined with specific growth factors, nutrients [7], and small molecules [6], resulting in improved hepatic functions in HLCs, evidenced by increased albumin secretion and cytochrome P450 enzyme 3A4 (CYP3A4) activity, as well as enhanced cell polarity [9, 10]. Materials such as Matrigel, laminin, and alginate are used to engineer 3D configurations like spheroids [11, 12], organoids [13, 14], and hydrogels for HLCs [15]. A previous investigation using an ECM microarray underscored the significance of both ECM composition and stiffness in facilitating the attachment of HLCs and promoting their differentiated functions; HLCs on a softer ECM scaffold displayed elevated albumin secretion and CYP3A4 activity within 2 weeks [16]. ECM–hepatocyte interactions are primarily mediated through integrin signaling that influences cell microstructure development, morphology, and behavior [17]. Although the function of HLCs was improved, it was still not comparable to PHHs, especially in terms of drug metabolism enzyme activities. Thus, it is pivotal to integrate ECM scaffolds with biochemical components encompassing essential elements of liver ECM composition, as well as controlling factors like topography, stiffness, and porosity, to study HLC differentiation.

Electrospinning has proven effective in producing porous nanoscale 3D fibers with substantial surface area, facilitating a high density of receptor ligands to modulate cellular phenotype [18]. Synthetic (e.g., polyethersulfone—PES, polycaprolactone—PCL) and natural polymers (gelatin, collagen, laminin, fibronectin, and decellularized liver ECM—dLECM) have all been employed for hepatocyte culture and functional analysis and highlighted the complex cell–ECM interactions present in the hepatic microenvironment [19]. For instance, collagen I-coated PES nanofibers were used for HLC differentiation, and they displayed hepatic gene expression (e.g., cytokeratin 19—*CK19*, albumin—*ALB*, α-fetoprotein—*AFP*, and *CYP7A1*) and albumin secretion [20, 21]. However, the mechanisms underlying the positive effects of ECM nanofibers on HLC differentiation need to be further elucidated.

In a previous study, we developed electrospun nanofibers from porcine liver extracellular matrix (PLECM) and chitosan, with or without type I collagen, and showed that these natural ECM-based nanofibers supported PHHs with higher and more stable hepatic functions (albumin and urea secretion, CYP450 activity) over several weeks in culture [18]. However, whether such nanofibers can drive the maturation of human iPSC-HLCs has remained unclear.

Here, we test the hypothesis that ECM composition, stiffness, and topography act in concert to promote functional maturation of HLCs. To do so, we fabricated nanofibers from rat-tail collagen I, PLECM, and chitosan, and benchmarked their effects against our prior PHH data [18]. In parallel, polyvinyl alcohol (PVA) was used as a stiff synthetic material to contrast with softer natural ECM nanofibers. Finally, we examined the role of Yes-associated protein 1 (YAP) signaling as a mechanistic link between ECM cues and HLC differentiation.

Overall, this study establishes a clear relationship between ECM biophysical and biochemical properties and HLC maturation, providing insights that are directly relevant to building more predictive platforms for drug screening, disease modeling, and regenerative medicine applications.

## 2 ∣ Materials and Methods

### 2.1 ∣ ECM Nanofiber Fabrication

Our previous publication detailed the procedures for collagen I extraction from rat tails and porcine liver decellularization [18]. The solvent 1,1,1,3,3,3-hexafluoro-2-propanol (HFIP) was used to dissolve collagen and PLECM blends overnight under constant stirring, while trifluoroacetic acid (TFA) was used to dissolve chitosan-only solutions. 5% (w/v) collagen and 2% (w/v) PLECM or chitosan were used to generate nanofibers. PVA was dissolved in deionized water at 5% (w/v) [22]. A glass syringe (Fortuna Optima, Luer lock tip style) containing the macromolecule solution was then placed in a Kent Scientific Genie Plus syringe pump (Torrington, CT), and the solutions were pumped (0.5–1.0 mL/h) for 5+ hours. A high-voltage DC power supply (Gamma High Voltage Research, Ormond Beach, FL), operating at 15–20 kV, created an electric field between a 19-gauge needle and a grounded copper collection plate covered with aluminum foil. Siliconized round glass coverslips (12 mm diameter, Hampton Research, Aliso Viejo, CA) were oxidized via oxygen plasma for 5 min to facilitate nanofiber attachment [23, 24] and then attached to the aluminum foil. The distance from the needle tip to the siliconized round glass coverslips was fixed at 15 cm for all conditions [22, 25]. Electrospinning was then conducted at 20°C ± 2°C and 19% relative humidity; electrospinning conditions for each macromolecule solution are provided in our previous publication [18].

Nanofibers that were adhered to the siliconized glass coverslips were crosslinked at room temperature (RT) for 24 h using a mixture of 1-ethyl-3-(3-dimethyl aminopropyl) carbodiimide (EDC, 20 mM) and *N*-hydroxysuccinimide (NHS, 10 mM) in 90% ethanol [26-28] for all nanofibers except for the chitosan-only and PVA nanofibers, which were cross-linked with a vapor of 25% glutaraldehyde in water and by heating at 180°C for 10 h, respectively. Lastly, the nanofibers were washed several times with sterile deionized water (obtained from a Milli-Q water purification system) and kept at 4°C until further use.

The O_2_ plasma-treated siliconized glass coverslips were incubated for 2 h in 50 µg/mL of Geltrex (Reduced-Growth Factor Basement-Membrane Matrix, Lactose Dehydrogenase Elevating Virus-free, stem-cell qualified, ThermoFisher, Waltham, MA) to generate control substrates. Following two rinses with double-distilled water (ddH_2_O), the coverslips were sterilized with 70% (v/v) ethanol in ddH_2_O for 1 h and then rinsed thrice with sterile ddH_2_O.

### 2.2 ∣ ECM Hydrogel Fabrication

Hydrogel preparation followed procedures in the literature with modifications [29, 30]. Briefly, solutions of 1% (w/v) collagen I and 1.5% (w/v) chitosan were prepared with 0.2 M hydrochloric acid (HCl) overnight at RT. The solutions were combined and stirred in a beaker with an ice water bath and a temperature of 1°C at a ratio of 1.4:1.0 (collagen:chitosan). After 5 min of stirring, EDC (66.2 mg) and NHS (39.8 mg) were added to the solution and stirred for 10 min. The pH of the solution was adjusted to 7 with 0.1 M sodium hydroxide (NaOH) solution. The hydrogels were pipetted (100 μL) on top of plasma-treated siliconized glass coverslips and incubated for 30 min at 37°C. After incubation, the hydrogels were dried overnight and sterilized with 70% ethanol for 30 min. Control chitosan/collagen “adsorbed” substrates were generated as above, except that no crosslinking reagents (EDC and NHS) were used.

### 2.3 ∣ Fiber Topography and Mechanical Characterization

Surface topography and mechanical properties of the nanofibers were obtained via atomic force microscopy (AFM) following previously published procedures [31, 32]. Briefly, AFM was performed on siliconized glass coverslips containing nanofibers, hydrogels, or adsorbed ECM. The surfaces were stuck to glass-bottom Petri dishes (Ted Pella #14036). Samples were imaged using the peak force QNM mode, using a BioScope Resolve BIOAFM (Bruker) with Nanoscope V controller. The measurements were performed under ambient conditions in 1× phosphate-buffered saline (PBS, Corning Life Sciences, Tewksbury, MA) at RT to characterize the morphology and the mechanical properties of the sample at the nanoscale in the hydrated state. The calibrated probe PF-QNM-LC-CAL with a tip radius of 65 nm was used, and deflection sensitivity was measured against a hard surface. The scan size was typically 5 × 5 μm^2^, with a digital resolution of 512 pixels × 512 pixels and a scanning rate of 0.8 Hz (lines per second). The oscillation frequency of the probe was set to 2 kHz, and the peak force set point was 1.5 nN. For every experiment, three measurements of Young's modulus on three different samples were acquired. Image analysis and nanomechanical property measurements were performed using NanoScope Analysis software (version 1.8), which automatically converted the Young's modulus measurements to stiffness values.

### 2.4 ∣ iPSC Culture and Hepatic Differentiation

Human K3 iPSCs were the gift of Dr. Stephen Duncan (Medical University of South Carolina) and derived from foreskin fibroblasts (CRL2097) obtained from ATCC [33, 34]. The iPSCs were differentiated into HLCs using previously published protocols with minor modifications [34-36]. Briefly, human iPSCs were seeded onto 50 μg/mL Geltrex-coated siliconized glass coverslips at a density of 7 × 10^5^ cells/mL to form a monolayer. Cells were induced to form definitive endoderm (DE) in RPMI 1640 Medium (Invitrogen, Waltham, MA) supplemented with 2% B-27 supplement minus insulin (Invitrogen), 10 ng/mL of bone morphogenetic protein 4 (BMP4) (Invitrogen), 20 ng/mL fibroblast growth factor 2 (FGF2) (Invitrogen), and 50–100 ng/mL Activin A (Invitrogen) for 2 days, followed by 50 ng/mL Activin A induction for 3 days. DE was then converted to specified hepatic endoderm by adding BMP4 (20 ng/mL) and FGF2 (10 ng/mL) for five additional days. Hepatocyte progenitor cells (HPCs) were generated by the inclusion of hepatocyte growth factor (HGF) (20 ng/mL) (Invitrogen) from days 10 to 15. Cells were induced to HLCs by adding oncostatin M (OSM) (20 ng/mL) (Invitrogen) for 5 days in hepatocyte culture medium (HCM) without epidermal growth factor (EGF) (Lonza, Walkersville, MD). Thus, based on this protocol, the iPSCs were considered HLCs after 20 total days of differentiation.

Nanofibers adhered to siliconized glass coverslips, and ECM adsorbed to siliconized glass coverslips were rinsed once with 1× PBS and incubated overnight in DMEM/F12 (ThermoFisher) containing 1% penicillin–streptomycin (Caisson Labs, Smithfield, UT). Then, they were transferred to the wells of a 24-well polystyrene plate pre-coated with 5% (m/v) Pluronic F-127 (Sigma-Aldrich) to prevent cell attachment to the polystyrene. Undifferentiated iPSCs were seeded onto the above substrates at 3.5–5 × 10^5^ cells in 500 μL per well of seeding medium containing mTeSR Plus (Stem Cell Technologies, Vancouver, BC) with 1% penicillin/streptomycin. The differentiation of iPSCs to DE cells, HPCs, and then HLCs on the nanofibers was the same as on the Geltrex-coated tissue culture-treated plate, as detailed above.

Most of the studies were conducted using the K3 iPSC line, except for specific validation studies with a second iPSC line, SCN5A1043, which was generated previously from peripheral blood mononuclear cells (PBMCs) by the Stanford Cardiovascular Institute [37, 38]. CHIR99021 (Sigma-Aldrich, Saint Louis, MO) at 3 μM was added on day 1 of differentiation for the SCN5A1043 iPSC line to improve HLC function [39, 40], but otherwise, the protocol for hepatic differentiation was identical to the K3 iPSC line, which did not benefit from inclusion of CHIR99021 (Figure S2G).

### 2.5 ∣ Cell Functional Assessments

Albumin in supernatants was measured using a sandwich-based enzyme-linked immunosorbent assay (ELISA, Bethyl Laboratories, Montgomery, TX) with horseradish peroxidase detection and 3,3′5,5′-tetramethylbenzidine substrate (TMB, Rockland Immunochemicals, Boyertown, PA). The absorbance of the samples was read on the synergy H1 multimode plate reader (Biotech, Winooski, VT). CYP3A4 enzyme activity was measured by incubating the cultures for 3 h with luciferin-IPA (Promega Life Sciences, Madison, WI), followed by the processing of collected supernatants per manufacturer's instructions; luminescence of the luciferase-treated luciferin was quantified with the synergy H1 multimode reader. The luminescence of the substrate incubated on an empty well (no cells) for the same duration was subtracted from the luminescence values obtained from the cells. The HLCs were not otherwise incubated with any CYP3A4-inducing drugs.

### 2.6 ∣ Microscopy for Cell Visualization

HLCs on nanofibers and siliconized glass controls were fixed with 4% (v/v) paraformaldehyde (PFA, Alfa Aesar, Wand Hill, MA) in ddH_2_O for 30 min, rinsed three times with 1× PBS, and then permeabilized with 0.5% (v/v) Triton X-100 (Ameresco, Solon, OH) in 1× PBS for 15 min. After washing with 1× PBS three times, cultures were incubated for 45 min at RT with a blocking solution containing 3% (w/v) bovine serum albumin (BSA) (Sigma-Aldrich). Goat anti-human albumin (Abcam, Cambridge, MA), rabbit anti-human cytokeratin 8 (CK8, Invitrogen, Waltham, MA), and mouse anti-human hepatocyte nuclear factor 4α (HNF4A, Santa Cruz Biotechnology, Dallas, TX) primary antibodies were diluted at 1:200 in dilution solution containing 1% (w/v) BSA (Sigma-Aldrich) in 1× PBS and incubated on the cultures at 4°C overnight. Cultures were rinsed the next day with 1× PBS thrice at 5 min per wash. Secondary antibodies, donkey anti-goat Alexa Fluor 488, donkey anti-mouse Alexa Fluor 647, and donkey anti-rabbit Alexa Fluor 568 (Invitrogen), were incubated at 1:200 dilution on cultures for 1 h at RT. DAPI (4′,6-diamidino-2-phenylindole, MP Biomedicals, Solon, OH) at 540 nM was added to the cultures for the last 15 min of the incubation period with the secondary antibodies. After incubation, the cultures were rinsed thrice with 1× PBS at 5 min per wash. Each nanofiber and siliconized glass control was mounted on a cover slide with a mounting medium (Biotium, Fremont, CA) for microscopic observations. iPSC-derived DE cells were stained using a similar protocol as above, except that the primary antibodies were mouse anti-human FOXA2 (Abnova, Taipei, Taiwan) and goat anti-human SOX17 (R&D Systems, Minneapolis, MN), diluted at 1:200 as mentioned above. The secondary antibodies and dilutions were the same as the ones above. DAPI at 300 nM was added to the cultures to stain nuclei.

Stained cultures were observed under confocal microscopy (Olympus/Evident Scientific FV3000, Tokyo, Japan) with a laser wavelength of 640 nm and filters of 650–750 nm for Alexa Fluor 647, a laser wavelength of 561 nm and filters of 570–620 nm for Alexa Fluor 568, a laser wavelength of 488 nm and filters of 500–540 nm for Alexa Fluor 488, and a laser wavelength of 405 nm with filters of 430–470 nm for DAPI. Total and HNF4A-positive cell numbers were quantified using CellProfiler.

For scanning electron microscopy (SEM), cultures were fixed for 45 min at RT with 3% (v/v) glutaraldehyde in ddH_2_O containing 0.1 M sucrose and 0.1 M sodium cacodylate (Sigma-Aldrich), followed by incubation for 10 min in a solution containing 0.1 M sodium cacodylate and 0.1 M sucrose. Samples were then dehydrated by adding increased ethanol concentrations (35%, 50%, 70%, and 100%, respectively) for 10 min each. Lastly, the samples were sputter-coated with gold (15 nm) and imaged using the JSM-6500F JEOL SEM (Tokyo, Japan) at an accelerating voltage of 15 kV.

### 2.7 ∣ Gene Expression Analysis

Cell samples were lysed with TRIzol (Invitrogen), and the RNA fraction was further isolated by introducing chloroform, centrifuging, and removing the top fraction. The isolated RNA samples were purified with the PureLink RNA Mini Kit (ThermoFisher). The genomic DNA was digested using amplification-grade DNase I (ThermoFisher) per the manufacturer's instructions. Purified RNA was then reverse transcribed into complementary DNA (cDNA) using the high-capacity cDNA reverse transcription kit (ThermoFisher) on a Veriti 96-well Fast Thermal Cycler (Applied Biosystems, Waltham, MA). About 1000 ng of cDNA was added to each quantitative polymerase chain reaction (qPCR) along with the SYBR green quantitative real-time polymerase chain reaction master mix (ThermoFisher) and pre-designed human primers (Table S1). These reactions were performed using the Cielo 3 qPCR system (Azure Biosystems, Dublin, CA). Target gene expression was first normalized to glyceraldehyde-3-phosphate dehydrogenase (*GAPDH*), which was shown to be a suitable reference gene for the human liver [41], and then to expression in a control condition [42].

### 2.8 ∣ Data Analysis

All findings were confirmed in 2–3 independent experiments (3–4 wells per condition and experiment). Data in each figure is shown from a single representative experiment with the K3 iPSC line, unless otherwise indicated. Data processing was performed using Microsoft Excel. GraphPad Prism software (La Jolla, CA) was used for data visualization and statistical analysis. Mean and standard deviation are displayed for all data sets. Statistical significance was assessed as follows: (1) comparisons between two groups were analyzed by two-tailed *t*-test; (2) comparisons among multiple groups were analyzed by one-way ANOVA with Dunnetťs multiple comparisons test (for comparisons to a single control) or with Bonferroni post hoc test (for all-group comparisons); and (3) comparisons among multiple groups with more than one independent variable were analyzed by two-way ANOVA with Dunnetťs multiple comparisons test or Bonferroni post hoc test. A *p* < 0.05 was considered statistically significant.

## 3 ∣ Results

### 3.1 ∣ Morphological and Mechanical Characterization of the ECM Nanofibers

**FIGURE 1 ∣:**
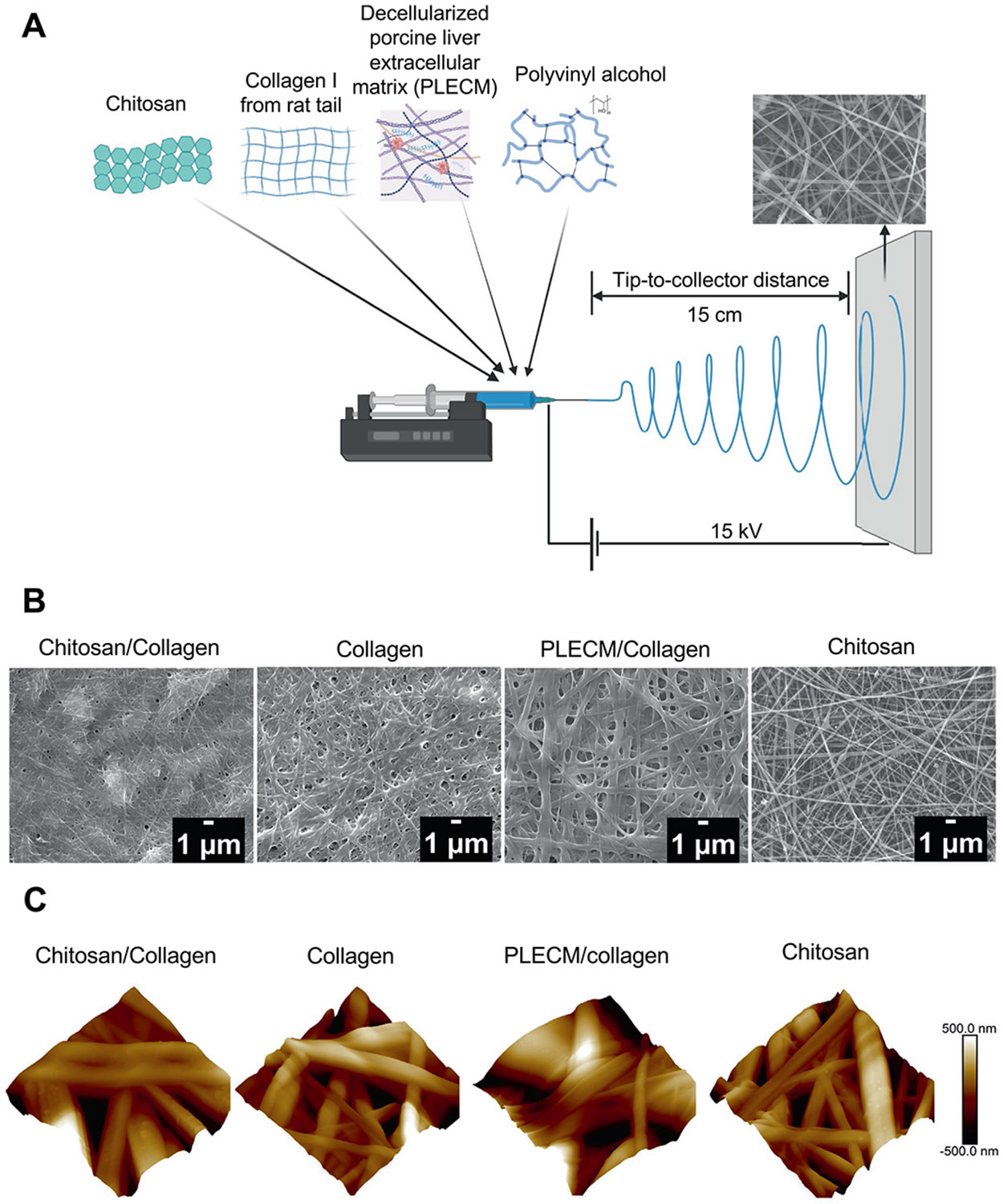
ECM nanofiber preparation and visualization. (A) HFIP was used to dissolve solutions containing rat tail collagen I or PLECM; TFA was used to dissolve the chitosan-only solution; deionized water was used to dissolve the PVA solution. Collagen was used at 5% (w/v) while PLECM and chitosan were used at 2% (w/v) each. All solutions were electrospun from the needle to an oxidized glass collector. Created with BioRender. (B) SEM images of spun nanofibers following hydration in culture medium. Scale bars are 1 μm. (C) AFM images of spun nanofibers. Scale represents the maximum and minimum height color on the AFM images.

Electrospun nanofibers were generated with (1) natural materials (rat tail collagen I, chitosan, chitosan/collagen, and PLECM/collagen) and (2) PVA (Figure 1A) [18]. All nanofibers showed similar diameters before (125–223 nm) and after (131–192 nm) crosslinking, as assessed via SEM (Figure 1B and Table S2). All nanofibers showed similar porosities before (49.4%–55.9%) and after (48.7%–53.3%) crosslinking, as well as similar pore areas before (0.27–0.58 μm^2^) and after (0.15–0.65 μm^2^) crosslinking (Table S3). The topography of the nanofibers was assessed via AFM (Figure 1C). Lastly, the average Young's modulus for the nanofibers was determined from AFM analysis and ranged from 1.75 to 94.4 MPa (Table 1). While the nanofibers composed of collagen alone or blended with chitosan and PLECM had similar Young's moduli (1.75–1.98 MPa), nanofibers containing chitosan alone had Young's moduli of 49.78 MPa, while PVA nanofibers were the stiffest at 94.4 MPa. The siliconized glass that was used for nanofiber deposition and Geltrex coating had a Young's modulus of ~11 MPa. The thickness of the nanofibers ranged from 101 to 178 μm, as measured using AFM, SEM, and caliper-based analysis.

### 3.2 ∣ iPSCs Can Be Differentiated Into DE Cells on ECM Nanofibers

**FIGURE 2 ∣:**
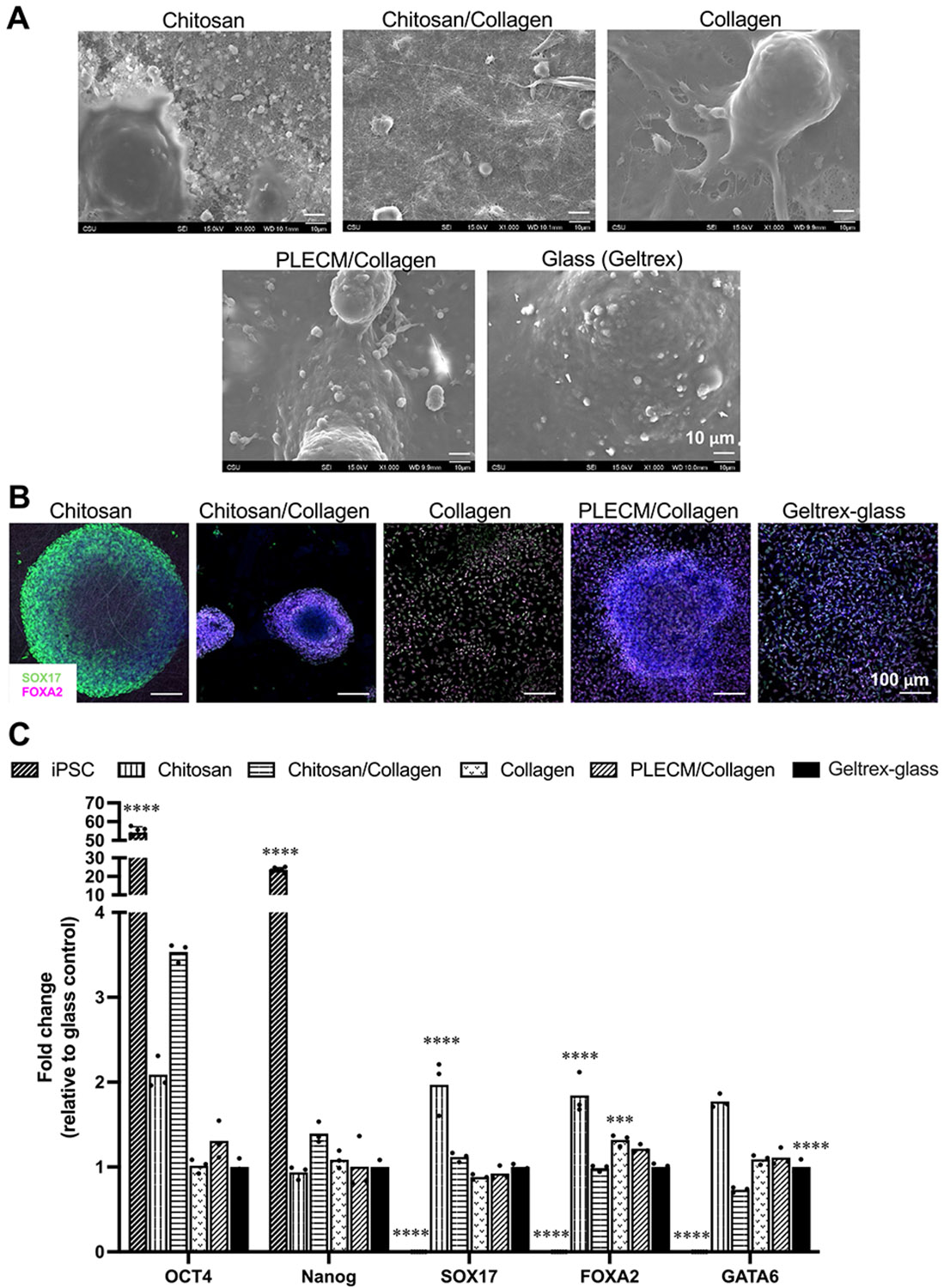
Morphology, staining, and gene expression of DE cells on ECM nanofibers. (A) SEM pictures of K3 iPSC-DE cells (5 days of differentiation) on the nanofibers and Geltrex-coated glass control. Scale bars are 10 μm. (B) Immunofluorescent staining of DE markers, SOX17 and FOXA2, on the various substrates. Scale bars are 100 μm. (C) Gene expression of DE cells on various substrates and undifferentiated iPSCs as a control (RNA from one well was analyzed in triplicate PCR reactions). Each gene's expression on the nanofiber substrates was normalized to GAPDH and then to the expression on Geltrex-glass. Statistical significance shown is relative to the Geltrex-glass control. Undifferentiated iPSCs were negative for the DE markers, *SOX17*, *FOXA2*, and *GATA6*, while stem cell pluripotency markers, *OCT4* and *Nanog*, were highly expressed in the undifferentiated iPSCs relative to the DE cells. Statistical significance was determined by one-way ANOVA with a Dunnett's multiple comparisons test. ***p <0.001, ****p <0.0001.

iPSCs were seeded onto the four types of ECM nanofibers mentioned above or Geltrex-coated siliconized glass coverslips as the conventional control; these cells were differentiated into DE cells for 5 days. While the size and spreading of DE cells varied on the various ECM substrates (Figure 2A), immunofluorescence revealed that all the ECM substrates promoted the expression of DE markers, SOX17 and FOXA2 (Figure 2B). The DE cells on chitosan and chitosan/collagen nanofibers formed cell aggregates, whereas they formed near-confluent monolayers on collagen nanofibers, PLECM/collagen nanofibers, and Geltrex-glass controls. Lastly, we used gene expression analysis to prove further that iPSCs differentiated into DE cells on nanofibers. Stem cell markers, *OCT4* and *Nanog*, decreased by 24–55-fold in DE cells across all ECM substrates as compared to undifferentiated iPSCs lysed immediately following their expansion on tissue culture plastic; in contrast, DE-specific markers, *SOX17*, *FOXA2*, and *GATA6*, were detectable in the DE cells on the various substrates after the 5 days of differentiation compared to the lack of expression in undifferentiated iPSCs (Figure 2C). Overall, chitosan-only nanofibers promoted higher expression of all three DE markers than the Geltrex-glass control, collagen nanofibers promoted higher expression of *FOXA2*, while DE marker expression on the chitosan/collagen and PLECM/collagen nanofibers was similar to the Geltrex-glass control.

### 3.3 ∣ ECM Nanofibers Promote a Higher Level of HLC Maturity

**FIGURE 3 ∣:**
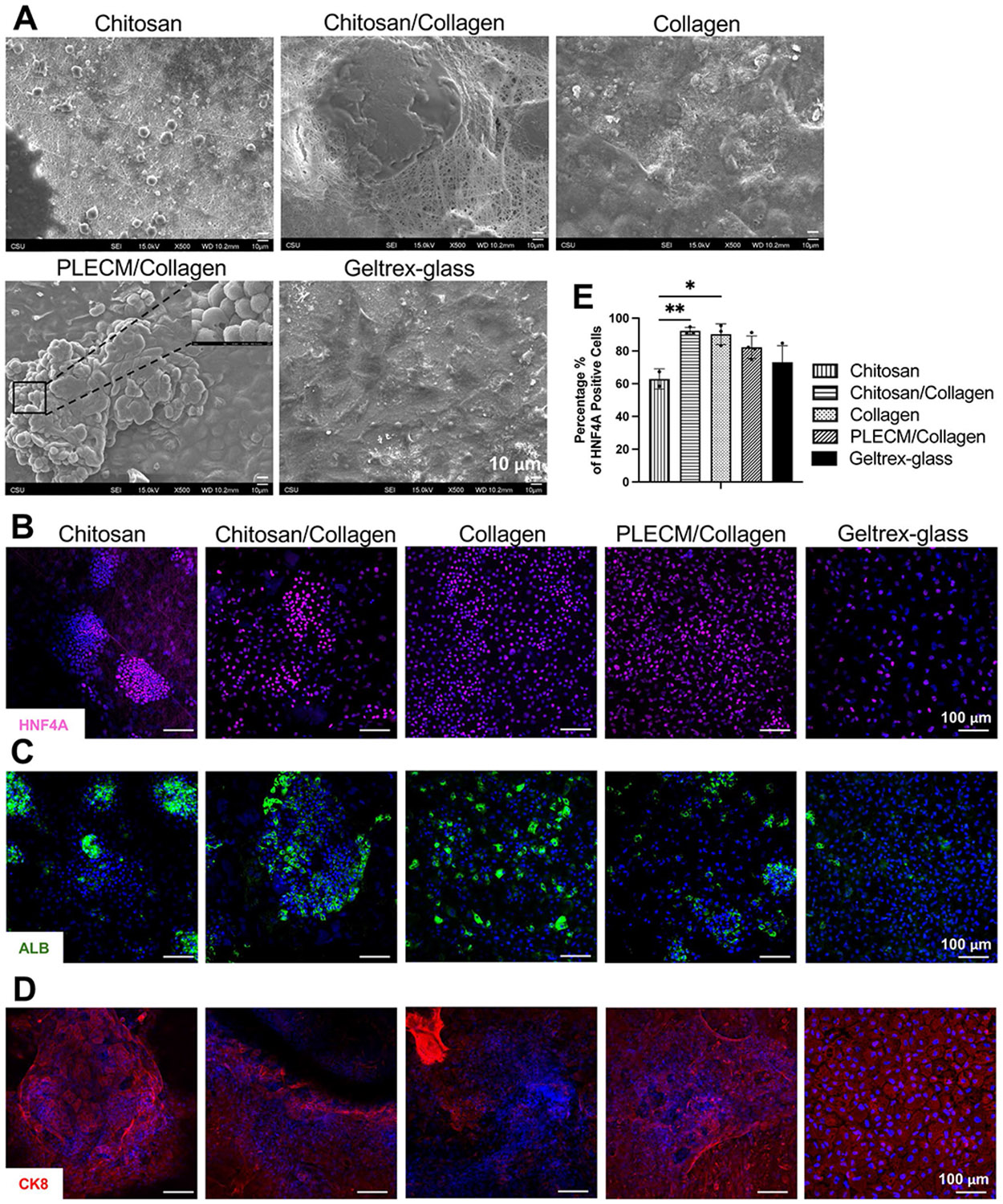
Morphology and staining of HLCs on ECM nanofibers. (A) SEM pictures of K3 iPSC-HLCs on the nanofibers and Geltrex-coated glass control (day 24). Scale bars are 10 μm. Immunofluorescent staining of HLC markers, (B) HNF4A (magenta), (C) ALB (green), and (D) CK8 (red) at day 24. Nuclei are counterstained with DAPI (blue). Scale bars for panels B–D are 100 μm. (E) The percentage of HNF4A-positive cells analyzed by CellProfiler (*n* = 2–4 images). Statistical significance was determined by one-way ANOVA with a Bonferroni post hoc test. *p < 0.05, **p < 0.01.

Next, iPSCs were differentiated into HLCs for at least 20 days. HLCs on chitosan, chitosan/collagen, and PLECM/collagen nanofibers formed cell aggregates, while they were more evenly spread on collagen nanofiber and Geltrex-coated glass control; furthermore, chitosan-only and PLECM/collagen nanofibers promoted a more 3D spherical HLC morphology (Figure 3A). Immunofluorescence revealed that HLCs formed clusters on chitosan and chitosan/collagen nanofibers (Figure 3B,C). Furthermore, HLC-specific markers, HNF4A (Figure 3B), ALB (Figure 3C), and CK8 (Figure 3D), were present across all substrates as assessed by immunofluorescence staining, albeit not all cells were positive for these markers, suggesting some undifferentiated cell types present even after 24 days of differentiation. Image analysis confirmed the qualitative observations in that 92% of the total number of DAPI-positive cells were also positive for HNF4A on chitosan/collagen nanofibers, 90% on collagen nanofibers, 82% on PLECM/collagen nanofibers, 73% on Geltrex-coated glass control, and 63% on the chitosan nanofibers (Figure 3E).

**FIGURE 4 ∣:**
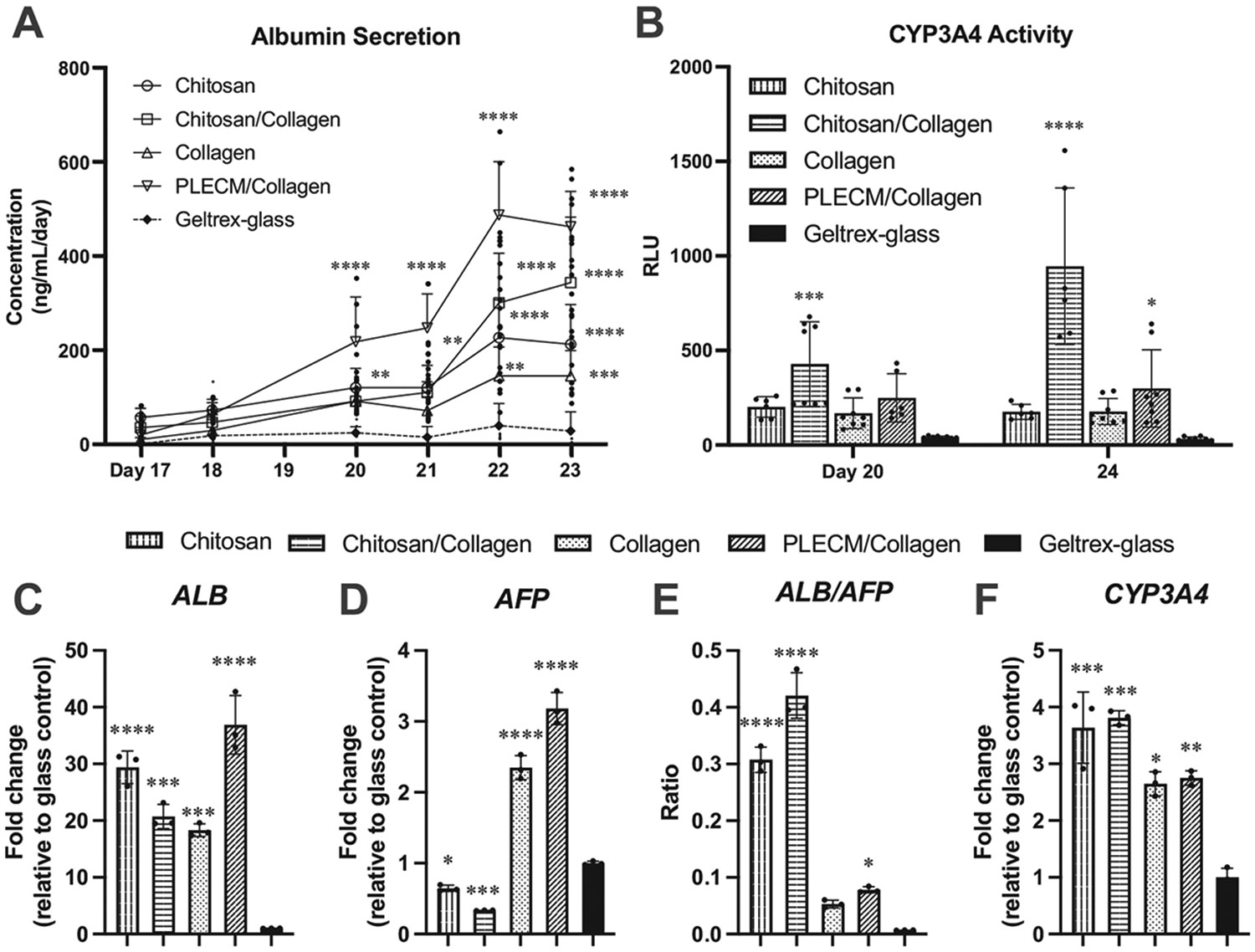
Functions and gene expression of HLCs on ECM nanofibers. (A) Albumin secretion and (B) CYP3A4 enzyme activities of K3 iPSC-HLCs on the ECM nanofibers and Geltrex-coated siliconized glass control (*n* = 3–4 replicate wells from the same experiment). Gene expression of (C) *ALB*, (D) *AFP*, (E) *ALB/AFP*, and (F) *CYP3A4* on the nanofibers and glass control at day 24 (RNA from one well was analyzed in triplicate PCR reactions). Each gene's expression on the nanofiber substrates was normalized to *GAPDH* and then to the expression on the Geltrex-glass control. Statistical significance is shown relative to the Geltrex-glass control in all panels. Statistical significance was determined by one-way (panels C–F) or two-way (panels A and B) ANOVA with a Dunnett's multiple comparisons test. *p < 0.05, **p < 0.01, ***p < 0.001, ****p <0.0001.

At the functional level, HLCs (K3 iPSC line) cultured on PLECM/collagen, chitosan/collagen, chitosan, and collagen nanofibers secreted ~16-, ~12-, ~7-, and ~5-fold more albumin than on the Geltrex-glass control, respectively (Figure 4A). In another differentiation batch, HLCs on chitosan, chitosan/collagen, PLECM/collagen, and collagen nanofibers secreted ~21-, ~17-, ~13-, and ~6-fold more albumin than on the glass control, respectively (Figure S1A).

The HLCs (K3 iPSC line) on the chitosan/collagen, PLECM/collagen, chitosan, and collagen nanofibers had ~23-, ~9-, ~5-, and ~5-fold higher CYP3A4 activity than on the Geltrex-glass control, respectively (Figure 4B). In another differentiation batch, HLCs on the chitosan, chitosan/collagen, PLECM/collagen, and collagen nanofibers had ~35-, ~27-, ~12-, and 6-fold higher CYP3A4 activity than on the Geltrex-glass control, respectively (Figure S1B).

Gene expression analysis confirmed the functional trends. The HLCs (K3 iPSC line) on the PLECM/collagen, chitosan, chitosan/collagen, and collagen nanofibers displayed ~37-, ~29-, ~22-, and ~18-fold higher *ALB* expression than on the Geltrex-glass control, respectively (Figure 4C). In another differentiation batch, the HLCs on chitosan, chitosan/collagen, PLECM/collagen, and collagen nanofibers displayed ~9-, ~4-, ~3.3-, and ~3.1-fold higher *ALB* expression than on the Geltrex-glass control, respectively (Figure S1C).

The expression of *AFP* (the fetal version of albumin) was also evaluated. HLCs (K3 iPSC line) on chitosan and chitosan/collagen nanofibers displayed lower *AFP* expression (0.6- and 0.3-fold, respectively), while the HLCs on collagen and PLECM/collagen nanofibers had higher *AFP* expression (2.3- and 3.2-fold, respectively) than the Geltrex-glass control (Figure 4D). However, when evaluating the *ALB/AFP* gene expression ratio, commonly used to assess the relative hepatic maturity, the HLCs on the chitosan/collagen, chitosan, PLECM/collagen, and collagen nanofibers had ~63-, ~45-, ~12-, and ~8-fold higher *ALB/AFP* ratio than on the Geltrex-glass control, respectively (Figure 4E). In another differentiation batch, the HLCs on chitosan and collagen nanofibers had similar, while chitosan/collagen and PLECM/collagen nanofibers had lower *AFP* expression (0.33- and 0.36-fold, respectively) than the Geltrex-glass control (Figure S1D). However, the HLCs on the chitosan, chitosan/collagen, PLECM/collagen, and collagen nanofibers had a 14-fold, ~11-, ~9-, and ~3-fold higher *ALB/AFP* gene expression ratio than on the Geltrex-glass control, respectively (Figure S1E).

Lastly, the HLCs (K3 iPSC line) on chitosan/collagen, chitosan, PLECM/collagen, and collagen nanofibers had ~3.8-, ~3.6-, ~2.7-, and ~2.6-fold higher *CYP3A4* expression than on the Geltrex-glass control, respectively (Figure 4F). In another differentiation batch, the HLCs on chitosan, chitosan/collagen, PLECM/collagen, and collagen nanofibers displayed ~6.2-, ~3-, ~2.6-, and ~2.1-fold higher expression than on the Geltrex-glass control, respectively (Figure S1F).

We also tested the effects of the nanofibers on HLCs derived from another iPSC line (SCN5A1043), although collagen-only nanofibers were not included, given their lowest effects on K3 iPSC-derived HLCs from the tested nanofibers as discussed above. The HLCs cultured on PLECM/collagen, chitosan, and chitosan/collagen nanofibers secreted ~1.8-, ~1.7-, and ~1.3-fold higher albumin than on the Geltrex-glass control by day 22, respectively, albeit the fold change on the chitosan-collagen nanofibers was not statistically significant (Figure S2A). The HLCs cultured on chitosan, PLECM/collagen, and chitosan/collagen nanofibers displayed ~7.9-, ~3.1-, and ~2.6-fold higher CYP3A4 activity than on the Geltrex-glass control, respectively, albeit only the fold change on the chitosan nanofibers was statistically significant (Figure S2B).

At the gene expression level, the HLCs (SCN5A1043 iPSC line) on chitosan/collagen, chitosan, and PLECM/collagen displayed ~3.6-, ~1.5-, and ~1.3-fold higher *ALB* expression than on the Geltrex-glass control, respectively (Figure S2C). The HLCs on chitosan/collagen nanofibers had ~3.5-fold higher *AFP* expression than on the Geltrex-glass control, while chitosan and PLECM/collagen nanofibers had lower (~0.5-fold) *AFP* expression than on the Geltrex-coated control (Figure S2D). The HLCs on the chitosan and PLECM/collagen nanofibers had a ~2.6- and ~2.4-fold higher *ALB/AFP* gene expression ratio than on the Geltrex-glass control, respectively, while the ratio on the chitosan/collagen nanofibers was nearly the same (Figure S2E). Lastly, the HLCs on chitosan/collagen, chitosan, and PLECM/ collagen displayed ~23.3-, ~6.3-, and ~6.1-fold higher *CYP3A4* expression than on the Geltrex-glass control, respectively (Figure S2F).

Overall, nanofibers consistently induced higher maturity in HLCs across multiple batches and two iPSC lines; however, the extent of maturity and the rank ordering of the nanofibers varied. Nonetheless, collagen-only nanofibers consistently performed the worst of those tested for the K3-HLCs (not tested for SCN5A1043). Furthermore, K3-HLCs functionally matured more on the nanofibers than SCN5A1043-HLCs.

### 3.4 ∣ ECM Nanofiber Topography Positively Influences HLC Maturity

**FIGURE 5 ∣:**
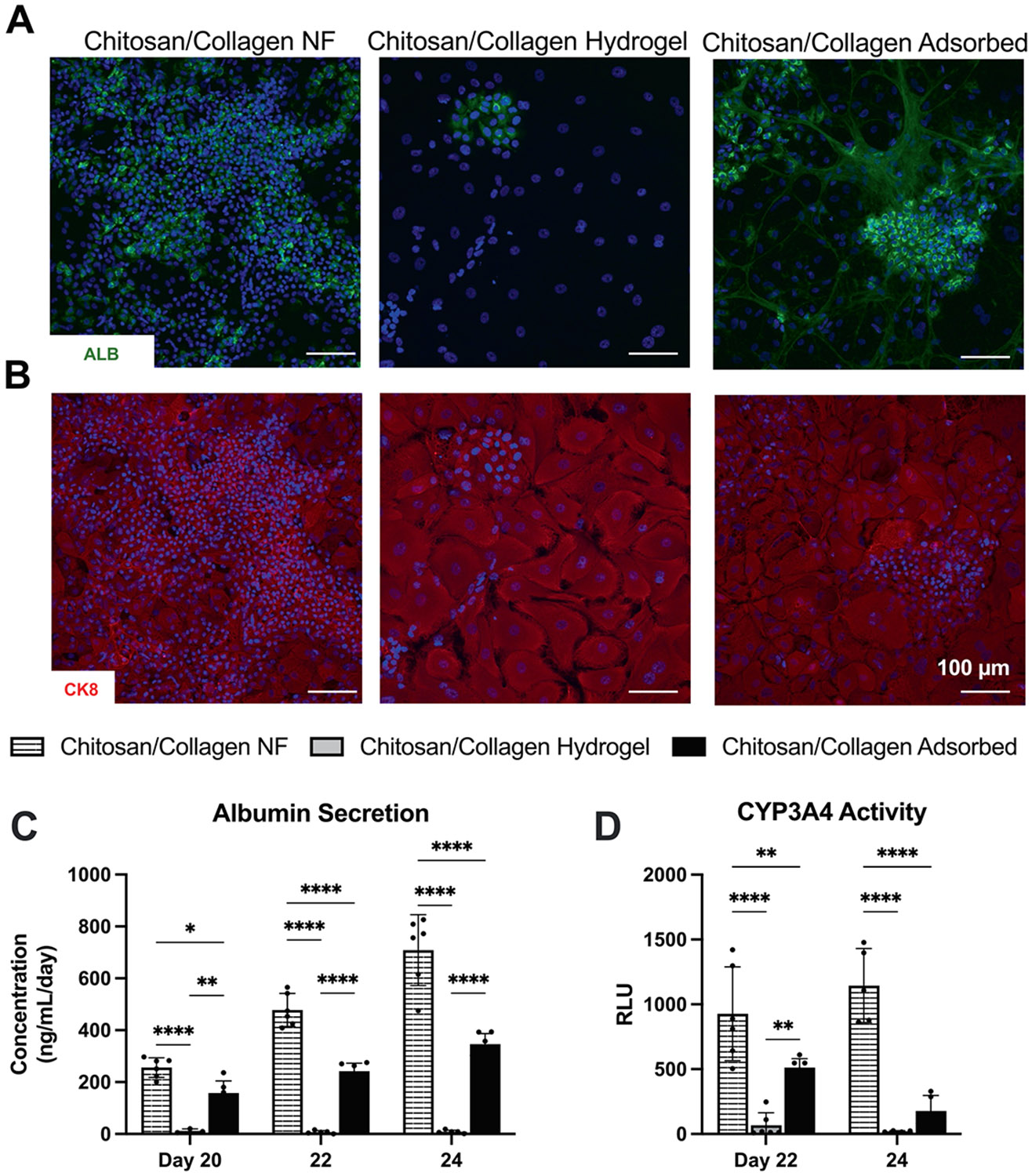
Effects of ECM nanofiber topography on HLC staining and functions. Chitosan/collagen was used to generate nanofibers (NF), a hydrogel (crosslinked), or adsorbed (not crosslinked) onto siliconized glass. K3 iPSCs were differentiated into HLCs on these ECM substrates. Immunofluorescent staining of HLC markers, (A) ALB (green) and (B) CK8 (red) at day 24. Nuclei are counterstained with DAPI (blue). Scale bars are 100 μm. (C) Albumin secretion and (D) CYP3A4 enzyme activities on ECM substrates (*n* = 3 replicate wells from the same experiment). Statistical significance was determined by two-way ANOVA with a Bonferroni post hoc test. *p < 0.05, **p <0.01, ****p <0.0001.

To elucidate the effects of nanofiber topography on HLC functions, we generated three substrate configurations using the chitosan/collagen blend on siliconized glass coverslips: nanofibers, hydrogels, and adsorbed ECM. The nanofibers and hydrogels had statistically similar Young's moduli (~0.9 to 1.8 MPa), which were lower than the adsorbed (uncrosslinked) ECM (~0.05 MPa) control (Table 1). Most HLCs were positive for CK8, while some were positive for albumin, as assessed via immunostaining (Figure 5A,B). At a functional level, the nanofibers led to the highest albumin secretion (Figure 5C) and CYP3A4 activities (Figure 5D) from the HLCs compared to the other two conditions. Interestingly, the hydrogel configuration was the lowest performing, with ~1% to 7% of albumin levels and ~1.7% to 13% of CYP3A4 activity detected in the other two conditions. The HLCs secreted between 1.6- and 2-fold higher albumin levels and displayed between 1.8- and 2.1-fold higher CYP3A4 activity on the chitosan/collagen nanofibers than the chitosan/collagen adsorbed control. These differences were lower than the 12-fold more albumin (Figure 4A) and 23-fold higher CYP3A4 activity (Figure 4B) detected from HLCs on the chitosan/collagen nanofibers compared to the Geltrex-glass control, thus suggesting that the more well-defined chitosan/collagen adsorbed coating may be more beneficial for HLC maturation than the poorly defined murine Engelbreth-Holm-Swarm tumor-derived Geltrex. However, the topography of the nanofibers led to higher HLC maturity; such was not entirely due to stiffness, since hydrogels of similar composition and stiffness were the worst performing of the three conditions tested.

### 3.5 ∣ ECM Stiffness Affects HLC Maturity and YAP-TAZ Signaling

**FIGURE 6 ∣:**
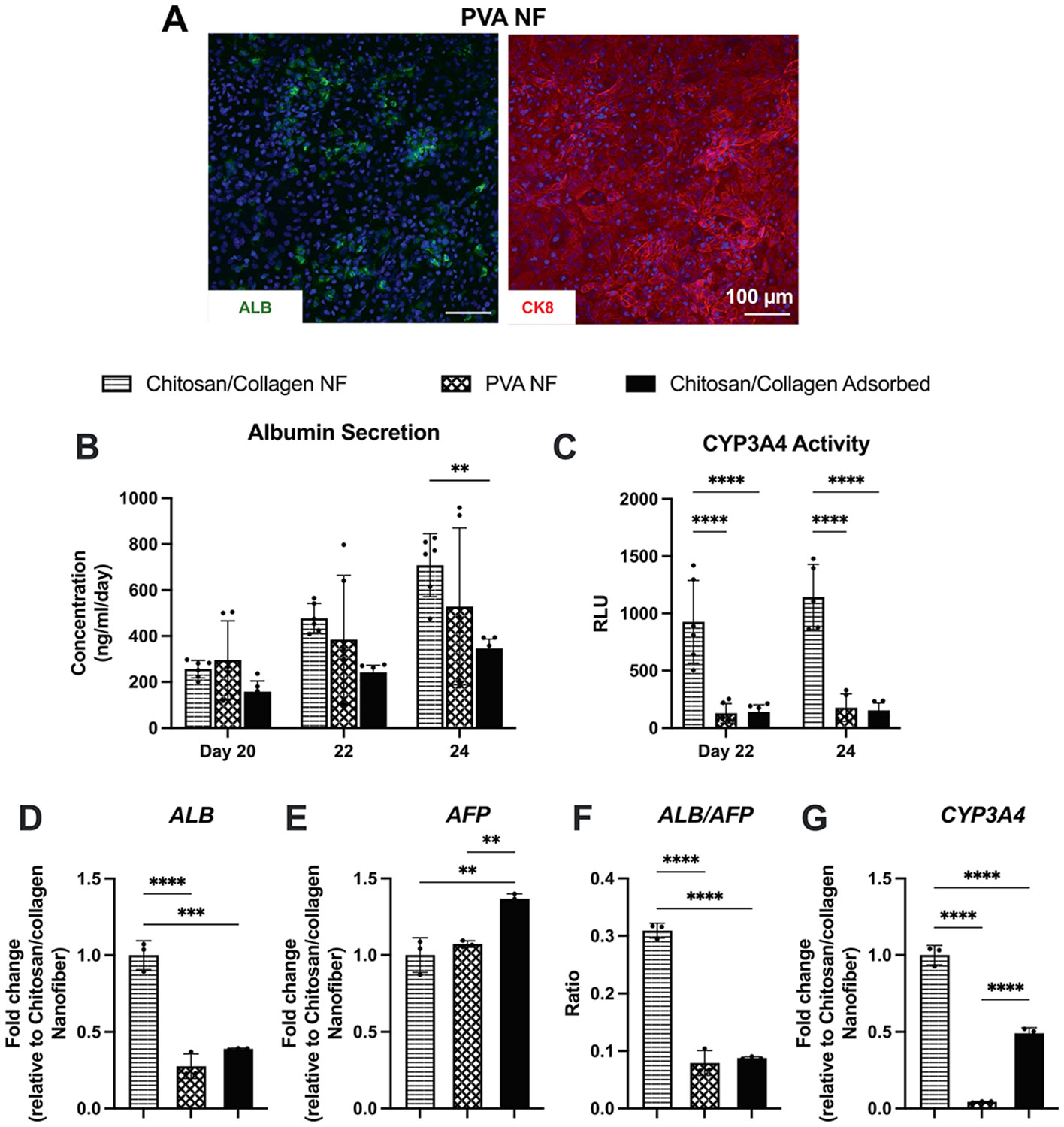
Effects of ECM nanofiber stiffness on HLC staining and functions. Chitosan/collagen and PVA were used to generate nanofibers (NF), while chitosan/collagen was adsorbed (not crosslinked) onto siliconized glass. K3 iPSCs were differentiated into HLCs on these ECM substrates. (A) Immunofluorescent staining of HLC markers, ALB (green), and CK8 (red) at day 24. Nuclei are counterstained with DAPI (blue). Scale bars are 100 μm. Similar staining for the other two substrates can be found in Figure 5A,B. (B) Albumin secretion and (C) CYP3A4 enzyme activities on ECM substrates (*n* = 3 replicate wells from the same experiment). Statistical significance was determined by two-way ANOVA with Bonferroni post hoc test. **p < 0.01, ****p < 0.0001. Gene expression of (D) *ALB*, (E) *AFP*, (F) *ALB/AFP*, and (G) *CYP3A4* in HLCs on the ECM substrates at day 24 (RNA from one well was analyzed in triplicate PCR reactions). Each gene's expression on the nanofiber substrates was normalized to *GAPDH* and then to the expression on the chitosan/collagen nanofibers. Statistical significance was determined by one-way ANOVA with a Bonferroni post hoc test. **p < 0.01, ***p <0.001, ****p <0.0001.

To elucidate the effects of nanofiber composition and stiffness on HLC functions, nanofibers of similar morphology and porosity were generated from PVA and chitosan/collagen blend; the chitosan/collagen adsorbed to siliconized glass was used as a control. The Young's modulus for chitosan/collagen nanofibers was 1.77 MPa, while that of PVA nanofibers was 94.4 MPa; the siliconized glass had a Young's modulus of 11.25 MPa (Table 1). Most HLCs were positive for CK8, while some were positive for albumin, as assessed via immunostaining (Figures 5A,B and 6A). At a functional level, the chitosan/collagen nanofibers led to the highest albumin secretion (Figure 6B) and CYP3A4 activity (Figure 6C) compared to the other two conditions. The PVA nanofibers had higher albumin secretion than the chitosan/collagen adsorbed control; however, the high variability of HLC response on PVA caused a lack of statistical significance compared to the chitosan/collagen adsorbed control. The CYP3A4 activity on PVA nanofibers and the chitosan/collagen adsorbed control was statistically similar.

Gene expression analysis confirmed the functional trends. *ALB* gene expression in HLCs was statistically similar on PVA nanofibers and the chitosan/collagen adsorbed control, and only ~23% to 37% of the gene expression detected on chitosan/collagen nanofibers (Figure 6D). The ECM adsorbed glass control had the highest *AFP* expression compared to chitosan/collagen and PVA nanofibers (Figure 6E). Additionally, the *ALB/AFP* gene expression ratio in HLCs (Figure 6F) was highest on the chitosan/collagen nanofibers, followed by statistically similar ratios on the PVA nanofibers and the chitosan/collagen adsorbed control (~22% to 28% of chitosan/collagen). Lastly, *CYP3A4* gene expression (Figure 6G) in HLCs was highest on the chitosan/collagen nanofibers, followed by the chitosan/collagen adsorbed control (~46.6% of chitosan/collagen), and the PVA nanofibers (~4%). Thus, natural chitosan/collagen nanofibers induced higher HLC maturation than synthetic and stiffer PVA nanofibers and the adsorbed ECM control.

**FIGURE 7 ∣:**
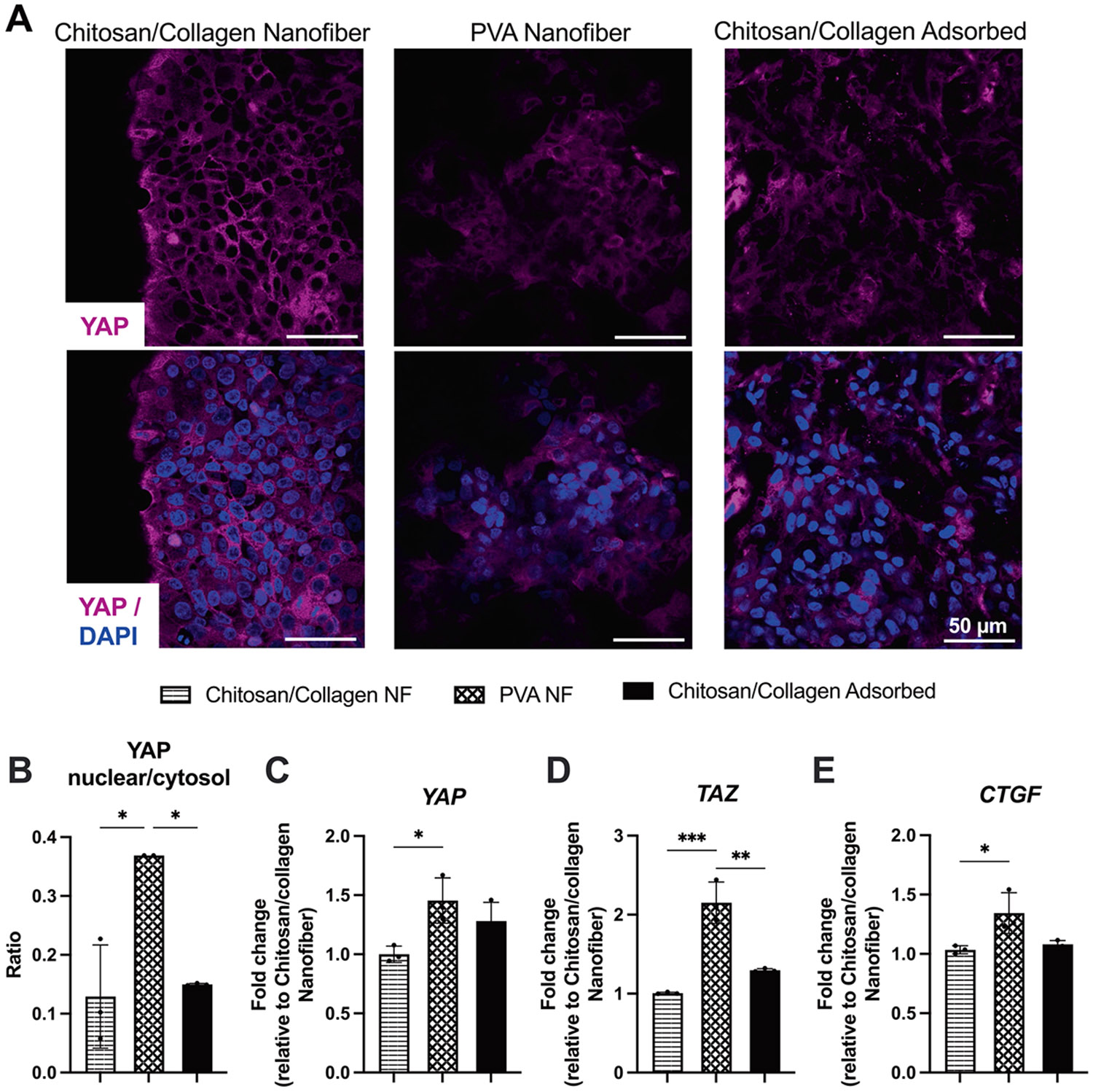
Effects of ECM nanofiber stiffness on YAP-TAZ signaling in HLCs. Chitosan/collagen and PVA were used to generate nanofibers (NF), while chitosan/collagen was adsorbed (not crosslinked) onto siliconized glass. K3 iPSCs were differentiated into HLCs on these ECM substrates. Immunofluorescent staining of (A) YAP and (B) YAP and DAPI overlap at day 24. Scale bars are 50 μm. (B) The ratio of YAP staining intensity in the nucleus: cytosol (multiple cells in *n* = 2–3 images quantified from technical replicate wells). Gene expression of (C) *YAP*, (D) *TAZ*, and downstream marker, (E) *CTGF* at day 24 (RNA from one well was analyzed in triplicate PCR reactions). Each gene's expression on the nanofiber substrates was normalized to *GAPDH* and then to the expression on the chitosan/collagen nanofiber. Statistical significance was determined by one-way ANOVA with a Bonferroni post hoc test. *p < 0.05, **p < 0.01, ***p < 0.001.

YAP-TAZ signaling is key in cellular sensing of the ECM's stiffness [43]. Therefore, we assessed such signaling in HLCs cultured on the chitosan/collagen nanofibers, PVA nanofibers, and chitosan/collagen adsorbed control. YAP immunostaining revealed that its nuclear to cytoplasmic ratio was ~3-fold higher in HLCs on the PVA nanofibers compared to chitosan/collagen nanofibers and chitosan/collagen adsorbed control, which had statistically similar ratios (Figure 7A,B). *YAP*, *TAZ*, and the downstream target gene, *CTGF* (Figure 7C-E), were also increased in HLCs on PVA nanofibers at the gene expression level compared to the other two conditions, which were statistically similar. Nuclear translocation of YAP enables it to function as a transcriptional co-activator, inducing genes like *CTGF* that mediate mechanosensing; in the context of hepatocytes, prior studies have shown that YAP nuclear activity suppresses hepatocyte differentiation [44, 45]. Thus, these results suggest that YAP-TAZ signaling is increased in HLCs on the PVA nanofibers and may explain the lower HLC maturity on the PVA nanofibers compared to the chitosan/collagen adsorbed on the softer siliconized glass and the softest chitosan/collagen nanofibers of the three substrates tested.

## 4 ∣ Discussion

Many studies of biomolecule-functionalized nanofibers have used synthetic polymers blended with tissue-specific ECM components; however, such a configuration does not fully mimic the ECM microenvironment of cells. In contrast, we previously developed a process to electrospin whole liver ECM and collagen I directly onto siliconized glass coverslips and then chemically crosslink the spun fibers for stability during long-term culture [18]. We also previously showed that PHH functions were enhanced for several weeks on the liver-like ECM nanofibers compared to the same materials adsorbed as a thin layer onto glass. However, PHHs are a limited resource for high-throughput drug screening, genotype–phenotype relationship assessment in disease modeling, and regenerative medicine. Such limitations are mitigated by iPSC-HLCs, but their functional maturation needs further improvement. Thus, here we investigated the effects of ECM nanofibers of different compositions on HLC morphology, staining patterns, functions, and gene expression over several weeks of culture; we further elucidated how ECM topography and stiffness regulate HLC functions and the involvement of YAP-TAZ signaling in such outcomes.

We chose four types of natural ECM nanofibers composed of rat tail collagen I alone, chitosan alone, chitosan/collagen, and PLECM/collagen; the ECM concentrations were chosen from our previous work for optimal electrospinning and to compare published PHH responses [18] to the HLC results obtained here. Rat tail collagen I and PLECM were selected over their human counterparts due to their cost-effectiveness for electrospinning and routine use and precedence for their use for human hepatocyte (primary and iPSC-derived HLC) culture [9, 18]; furthermore, obtaining human LECM of consistent quality from transplant-rejected human livers of variable conditions is not trivial. Chitosan is often incorporated into hydrogels to enhance their mechanical strength [30], and collagen/chitosan hydrogels have demonstrated strong cell adhesion and proliferation for fibroblasts [30], endothelial cells [29], and in vivo applications [46]. However, we did not select a PLECM-only solution for HLC culture as we previously found that it generates microfibers instead of nanofibers, the mechanism of which is not yet known [18].

The Young's moduli of all the nanofibers were statistically similar (~2 MPa), except for the much stiffer chitosan-only nanofibers (~50 MPa). While the stiffness of the nanofibers is higher than that of native liver (~0.5 to 4 kPa) [47, 48], it is still three orders of magnitude lower than glass (GPa). Furthermore, these nanofibers maintained statistically similar diameter ranges before and after crosslinking (~200 nm), average porosities (~50%), and average pore areas (~1 μm^2^); such similarities in biophysical parameters allowed us to evaluate the effects of biochemical composition on HLC functions. Lastly, we generated HLCs on a Geltrex-coated (extracted in the same way as Matrigel) 2D surface as a conventional control utilized in the field [36].

We found that iPSCs adhered to the above ECM nanofibers and differentiated into DE cells over 5 days and then into HLCs over the subsequent 15 days with the supplementation of culture medium with the relevant differentiation factors. All ECM nanofibers promoted the expression of DE markers. Chitosan and chitosan/collagen nanofibers promoted the formation of cell aggregates, while collagen and PLECM/collagen nanofibers promoted confluent monolayer formation similar to the Geltrex-glass control. This outcome may be because chitosan lacks any proteins that might mimic liver-like ECM proteins, which is mitigated to some extent by blending it with collagen I. Interestingly, chitosan-only nanofibers promoted the highest expression of DE markers, likely due to the promotion of a more 3D morphology, which has been previously shown to promote iPSC to hepatocyte differentiation [49]. As with DE cells, HLCs formed aggregates/clusters on chitosan and chitosan/collagen nanofibers. Additionally, > 80% of HLCs were positive for the master liver transcription factor, HNF4A, on all nanofibers except for the chitosan nanofibers (63%) and the Geltrex-glass (73%) control.

HLCs on all nanofibers across two iPSC lines displayed higher maturity than on the Geltrex-glass control. However, the fold induction was higher for the K3 line compared to the SCN5A1043 line. For instance, albumin secretion in K3-HLCs was enhanced ~5- to 21-fold on nanofibers, whereas it was enhanced ~1.3- to 1.8-fold in SCN5A1043-HLCs. Similarly, CYP3A4 activity in K3-HLCs was enhanced ~5- to 35-fold on nanofibers, whereas it was enhanced ~2.6- to 8-fold in SCN5A1043-HLCs. While the mechanisms underlying the functional differences in the differentiation of each iPSC line to HLCs on the nanofibers are unclear and require further experimentation, it is not uncommon for different iPSC lines to respond variably to in vitro stimuli [16]. Such variable responses could potentially be due to any residual epigenetic memory left in each iPSC line from their original somatic source (e.g., foreskin fibroblasts for K3 and PBMCs for the SNC5A1043 line) that predisposes them to different extents of signaling response to the administered differentiation soluble factors in the culture medium. Ultimately, further standardization of iPSC line generation and concentrations of differentiation factors will be necessary in the field to reduce variability in the maturation response to differentiation cues, including ECM nanofibers. Nonetheless, since CYP3A4 constitutes ~30% to 40% of all CYP450 isoforms in the adult liver, it metabolizes ~50% of marketed drugs, and its gene expression and activity are generally very low (< 5% of PHH levels) in HLCs differentiated using conventional protocols [49, 50], the improvement observed here with the nanofibers is highly encouraging.

Another method to assess HLC maturity is by evaluating the ALB to AFP (fetal form of ALB) ratio, which increases with maturity as HLCs upregulate albumin production and down-regulate AFP [49]. The K3-HLCs displayed ~3- to 63-fold higher *ALB/AFP* gene expression ratio, while the SCN5A1043-HLCs displayed up to ~2.6-fold higher *ALB/AFP* compared to the Geltrex-glass control, which, when coupled with the functional data, suggests that K3-HLCs functionally matured significantly more on the ECM nanofibers than the SCN5A1043-HLCs.

Across multiple batches of the K3-HLCs, the collagen nanofibers upregulated HLC maturity compared to the Geltrex-glass control but were the worst-performing across the different nanofiber types tested here; therefore, they were not tested on the SCN5A1043-HLCs, which were tested with chitosan, chitosan/collagen, and PLECM/collagen nanofibers. While chitosan nanofibers induced high levels of functional maturity in HLCs derived from both iPSC lines, the inclusion of collagen with chitosan (i.e., chitosan/collagen) led to more consistent maturation across different experiments, likely due to signaling from the collagen as the iPSCs matured toward the HLC phenotype. Lastly, the PLECM/collagen nanofibers also induced a high level of maturity in HLCs, but on average, they induced lower functions than the chitosan-containing nanofibers.

When comparing our previously published PHH monoculture responses on the same ECM nanofibers [18], we noticed key differences with our HLC findings here. First, relative to a conventional control for each cell type, ECM nanofibers induced greater albumin secretion in HLCs (up to 21-fold) than in PHHs (up to ~5-fold); similarly, nanofibers induced up to 35-fold CYP3A4 activity in HLCs compared to ~4-fold in PHHs. Second, while chitosan/collagen nanofibers induced some of the lowest functions in PHHs, they induced some of the highest functions in HLCs. In contrast, while collagen nanofibers induced some of the highest functions in PHHs, they induced some of the lowest in HLCs, albeit still higher than the Geltrex-glass control. HLCs may secrete their own ECM proteins to modify the coating of the chitosan/collagen nanofibers, which also concurrently promote more aggregation of the HLCs as observed here. Third, the maximal CYP3A4 activity measured in HLCs cultured on the nanofibers was ~19% of the activity in PHHs cultured on the nanofibers. Thus, HLCs, even on nanofibers, are not quite as mature as their PHH counterparts cultured in the same way, although the functional improvement compared to the Geltrex-glass control is significant, as quantified above. We anticipate that co-culture with liver non-parenchymal cells will be necessary to further mature the HLCs on the nanofibers, as we have previously observed with micropatterned HLC-fibroblast co-cultures on adsorbed collagen I plastic [9] and with PHH-fibroblast co-cultures on ECM nanofibers [18].

To isolate the effects of ECM topography from stiffness on HLCs, we generated chitosan/collagen hydrogels of a statistically similar stiffness (Young's modulus ~0.9 to 1.8 MPa) as the chitosan/collagen nanofibers; we also absorbed the chitosan/collagen mixture onto the glass without crosslinking it, and thus it had a Young's modulus of ~0.05 MPa. The nanofibers induced the highest HLC maturity (albumin secretion and CYP3A4 activity) of the three substrates, about twofold higher than the adsorbed chitosan/collagen substrate. The next highest induction was achieved with the adsorbed chitosan/collagen substrate, which promoted higher albumin production and CYP3A4 activity than the murine tumor-derived Geltrex-glass. Interestingly, the chitosan/collagen hydrogel configuration performed the lowest in all three conditions (~1% to 13% of HLC functions). Given the increased spreading of HLCs and what appeared like fewer numbers from immunostained images on the hydrogels compared to the other two conditions, we suspect that a soft but relatively smooth substrate is not entirely conducive to cell growth and migration as iPSCs differentiate into HLCs, likely due to the formation of fewer focal adhesions; however, proving this hypothesis will require further experimentation in the future. Lastly, various signaling pathways (e.g., Wnt signaling) are implicated in topography-driven cell differentiation, which can be the subject of future investigations on the nanofiber platform [51].

Since we showed that ECM topography improves HLC maturation, we next isolated stiffness by generating PVA nanofibers of similar diameters as the chitosan/collagen nanofibers, except much stiffer (~1.8 vs. 94 MPa), as expected from previous literature [22]. HLCs secreted more albumin on the chitosan/collagen than on the PVA nanofibers, although statistical significance was not reached due to the higher variability in the HLC response on the PVA nanofibers. At the gene expression level, HLC *ALB* on PVA nanofibers was only 22% of the chitosan/collagen nanofibers, and such was statistically significant. CYP3A4 activity and *CYP3A4* gene expression were ~7- and ~25-fold higher, respectively, on the chitosan/collagen nanofibers compared to the PVA nanofibers. Similarly, the *ALB/AFP* gene expression ratio in HLCs was ~4-fold higher on the chitosan/collagen nanofibers than on the PVA nanofibers. Except for albumin secretion, all other markers above were similar or lower in HLCs on the PVA nanofibers than on the chitosan/collagen adsorbed to the softer (11.25 MPa) siliconized glass. These results show that a lower stiffness and biochemical composition synergize to positively modulate HLC gene expression and functions, especially CYP3A4 expression and activity.

While PVA lacks biochemical signals that could induce a high level of HLC maturity, scaffolds fabricated using PVA offer superior mechanical stability, encompassing high tensile strength, elongation at break, and gradual degradation kinetics compared to natural nanofibers [52]. They are also biocompatible, biodegradable, transparent, thermo-stable, and chemically resistant, which has made them useful for drug delivery in vivo and reinforcing other tissues such as bone, cartilage, and skin [53-56]. While beyond the scope of this work, mixing PVA with chitosan and collagen to generate nanofibers may allow for the incorporation of desirable properties of the different ECM types for HLC maturation for both in vitro and in vivo applications.

Cells sense and transduce mechanical forces, such as stiffness, shear stress, and compression stress, into biochemical signals by activating specific genes and signaling cascades to adapt to the physical microenvironment. The Hippo pathway is one of the most significant signaling pathways that regulates YAP and TAZ proteins, which govern the behavior of cells in response to mechanical stimuli, such as ECM stiffness [57]. On a softer substrate, integrin α/β on the cell surface recruits FAK/SRC, which leads to the activation of RAP2 and eventually promotes YAP phosphorylation and inactivation; in contrast, stiffer substrates impede RAP2 activation and thus facilitate YAP's nuclear translocation [58, 59]. Prior studies have shown that increased YAP nuclear activity can cause hepatocyte de-differentiation by repressing HNF4A and promoting differentiation into cholangiocytes [44, 45]. Here, we found that the gene expression of *YAP*, *TAZ*, and a downstream marker, *CTGF*, increased in HLCs while YAP protein translocated to the nucleus at a higher level on the stiffest (~94 MPa) PVA nanofibers than on the softer (~0.94 MPa) chitosan/collagen nanofibers. However, interestingly, such a trend was not observed in HLCs cultured on chitosan/collagen adsorbed to siliconized glass, which is stiffer (~11 MPa) than the chitosan/collagen nanofibers but softer than the PVA nanofibers. Thus, these results suggest that there is likely a stiffness threshold that causes detectable increases in the YAP nuclear to cytoplasmic ratio and downstream gene expression.

In conclusion, we show that the topography and biochemical composition of electrospun natural ECM nanofibers can synergize to improve the maturation of iPSC-HLCs, partly by suppressing YAP localization to the nucleus, as compared to conventional Geltrex-glass control and stiff synthetic PVA nanofibers. The ECM nanofiber platform can ultimately prove useful for drug toxicity screening, disease modeling, and to inform design choices for vascularized liver surrogates for regenerative medicine.

## Supplementary Material

Supplemental Data

Additional supporting information can be found online in the Supporting Information section. Data S1: Supplementary Information

## Figures and Tables

**TABLE 1 ∣:** Mechanical properties of ECM substrates used in this study.

ECM substrate	Young's modulus (MPa)	Log (modulus)	Stiffness (N/m)
Chitosan nanofibers	49.78 ± 18.76	7.67 ± 0.15	1.07 ± 0.30
Collagen I nanofibers	1.98 ± 0.56	6.28 ± 0.13	0.09 ± 0.02
Chitosan/collagen I nanofibers	1.77 ± 0.90	6.19 ± 0.24	0.09 ± 0.04
PLECM/collagen I nanofibers	1.75 ± 0.94	6.19 ± 0.23	0.10 ± 0.04
PVA nanofibers	94.40 ± 32.11	7.95 ± 0.17	1.62 ± 0.47
Chitosan/collagen hydrogel (crosslinked)	0.94 ± 0.27	5.96 ± 0.14	0.05 ± 0.01
Collagen/chitosan adsorbed (not crosslinked) onto glass	0.05 ± 0.03	4.64 ± 0.28	0.01 ± 0.003
Siliconized glass	11.25 ± 4.87	7.00 ± 0.19	0.24 ± 0.07

*Note: N* = 3 for all data.

## Data Availability

The data that support the findings of this study are available from the corresponding author upon reasonable request.
