# Supplementary material for: Biochemical and Biophysical Properties of Extracellular Matrix Nanofibers Modulate iPSC-Derived Human Hepatocyte Maturation
Source: J Biomed Mater Res A. Author manuscript; Available in PMC 2026 Jul 21. (PMC13385130; doi:10.1002/jbm.a.37998)
Supplement: Supplemental Data [file NIHMS2192760-supplement-Supplemental_Data.pdf]

Supplemental Material

Biochemical and Biophysical Properties of Extracellular Matrix Nanofibers Modulate iPSC-derived Human Hepatocyte Maturation by *Yuan, Y. et al.*

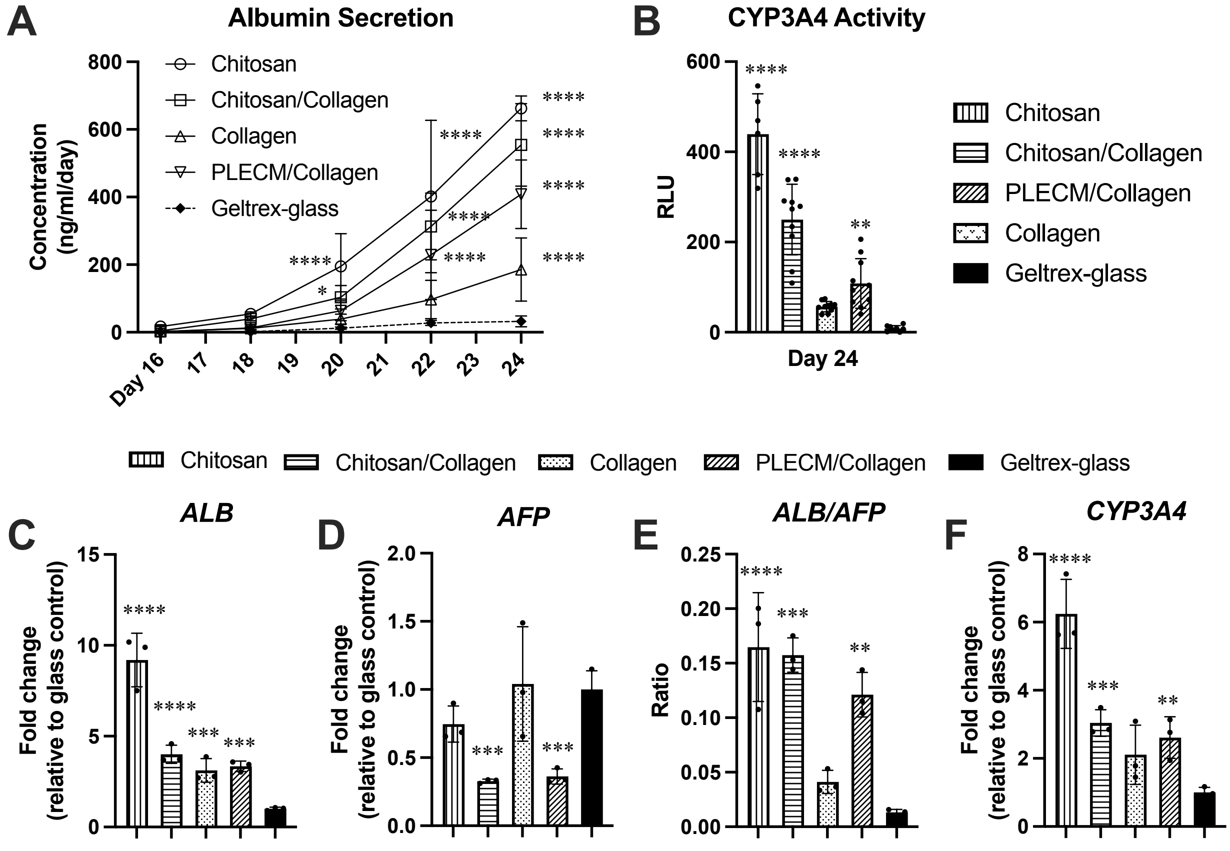

Supplemental Figure 1. Functions and gene expression of K3 iPSC-derived HLCs on ECM nanofibers (a different differentiation batch than shown in Figure 4 of the main manuscript). (A) Albumin secretion and (B) CYP3A4 enzyme activities of K3 iPSC-derived HLCs on the ECM nanofibers and Geltrex-coated siliconized glass control (n=3-4 replicate wells from the same experiment). Gene expression of (C) *ALB*, (D) *AFP*, (E) *ALB/AFP*, and (F) *CYP3A4* on the nanofibers and glass control at day 24 (RNA from one well was analyzed in triplicate PCR reactions). Each gene's expression on the nanofiber substrates was normalized to *GAPDH* and then to the expression on the Geltrex-glass control. Statistical significance is shown relative to the Geltrex-glass control in all panels. Statistical significance was determined by one-way (panels C-F) or two-way (panels A-B) ANOVA with a Dunnett's multiple comparisons test. *p<0.05, **p<0.01, ***p<0.001, ****p<0.0001.

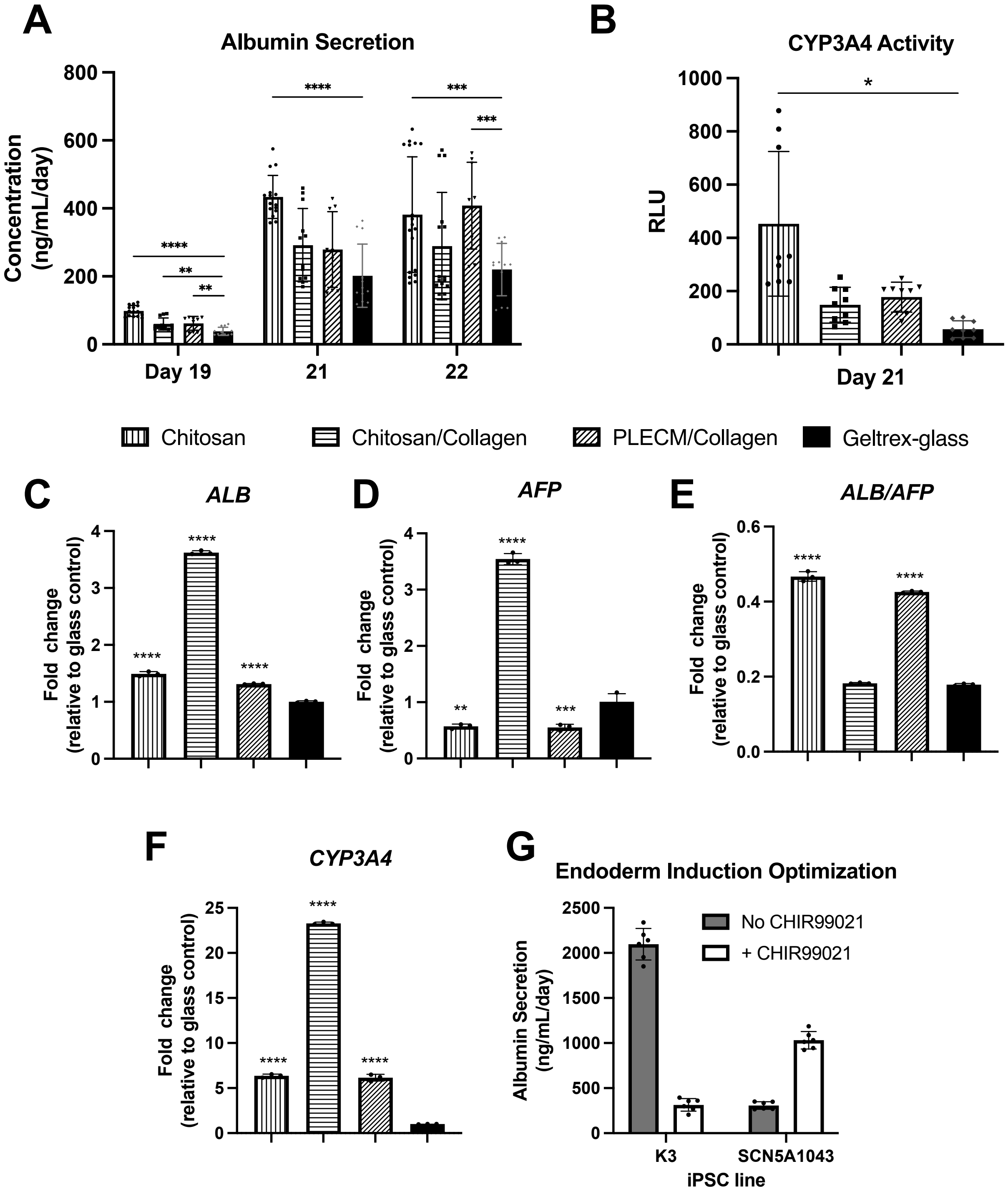

Supplemental Figure 2. Functions and gene expression of SCN5A1043 iPSC-derived HLCs on ECM nanofibers. (A) Albumin secretion and (B) CYP3A4 enzyme activity of HLCs on the ECM nanofibers and Geltrex-coated siliconized glass control (n=9-18 replicate samples from the same experiment). Gene expression of (C) *ALB*, (D) *AFP*, (E) *ALB/AFP*, and (F) *CYP3A4* on the nanofibers and glass control at day 22 (n=3 replicate samples from the same experiment). Each gene's expression on the nanofiber substrates was normalized to *GAPDH* and then to the

expression on the Geltrex-glass control. Statistical significance is shown relative to the Geltrex-glass control in all panels. Statistical significance was determined by one-way (panels C-F) or two-way (panels A-B) ANOVA with a Dunnett's multiple comparisons test. *p<0.05, **p<0.01, ***p<0.001, ****p<0.0001. (G) Albumin secretion on day 20 of differentiation for HLCs generated from the two indicated iPSC lines, where the lines were either treated or not with CHIR99021 on day 1 of differentiation.

SUPPLEMENTAL TABLES

Gene	Forward Primer	Reverse Primer
AFP	CTG CAG CCA AAG TGA AGA GGG AAG	GTA GGT GCA TAC AGG AAG GGA TGC
ALB	GCT GCC ATG GAG ATC TGC TTG AAT	GCA AGT CAG CAG GCA TCT CAT CAT
CTGF	TGC CCT CGC GGC TTA CCG ACT G	TGC AGG AGG CGT TGT CAT TGG TAA C
CYP3A4	TTT ATG ATG GTC AAC AGC CTG TGC	CTG GTG AAG GTT GGA GAC AGC AAT
FOXA2	GGG AGC GGT GAA GAT GGA	TCA TGT TGC TCA CGG AGG AGT A
GAPDH	AGC CAC ATC GCT CAG ACA CC	GTA CTC AGC GCC AGC ATC G
GATA6	CCA TCT TGA CCC GAA TAC TTG A	GCA AAA ATA CTT CCC CCA CAA C
NANOG	CCT GTG ATT TGT GGG CCT GAA GAA	GCA GAA GTG GGT TGT TTG CCT TTG
OCT4/POU5F1	GGA GAA GGA GAA GCT GGA GCA AAA C	TAT CCC AGG GTG ATC CTC TTC TGC
SOX17	CCA AGG GCG AGT CCC GTA TC	CAC GAC TTG CCC AGC ATC TTG
YAP	TCC CAG CAC AGC AAA TTC TCC AAA	AAT TCC TGA GAC ATC CCG GGA GAA

Supplemental Table 1: List of PCR primers used in this study.

Sample	Diameter, un-crosslinked (nm)	Diameter, crosslinked (nm)
Chitosan nanofibers	175 ± 87	178 ± 85
Chitosan/Collagen nanofibers	125 ± 52	131 ± 51
PLECM/Collagen nanofibers	223 ± 129	192 ± 83
Collagen nanofibers	187 ± 86	165 ± 70

Supplemental Table 2: Diameter range of nanofibers. N=3 for all samples.

Sample	Uncrosslinked		Crosslinked	
	Porosity (%)	Pore area (μm^2)	Porosity (%)	Pore area (μm^2)
Chitosan nanofibers	52.8	0.38 ± 0.51	48.8	0.30 ± 0.40
Chitosan/Collagen nanofibers	55.9	0.27 ± 0.50	53.3	0.15 ± 1.12
PLECM/Collagen nanofibers	49.4	0.58 ± 0.83	48.7	0.65 ± 1.39
Collagen nanofibers	49.9	0.39 ± 0.58	51.0	0.27 ± 1.00

Supplemental Table 3: Porosity and pore size range of nanofibers. N=3 for all samples.
